# Proton Radiation Effects on Dark Signal Distribution of PPD CMOS Image Sensors: Both TID and DDD Effects

**DOI:** 10.3390/s17122781

**Published:** 2017-11-30

**Authors:** Yuanyuan Xue, Zujun Wang, Wei Chen, Minbo Liu, Baoping He, Zhibin Yao, Jiangkun Sheng, Wuying Ma, Guantao Dong, Junshan Jin

**Affiliations:** State Key Laboratory of Intense Pulsed Irradiation Simulation and Effect, Northwest Institute of Nuclear Technology, Xi’an 710024, China; xueyuanyuan@nint.ac.cn (Y.X.); liuminbo@nint.ac.cn (M.L.); hebaoping@nint.ac.cn (B.H.); yaozhibin@nint.ac.cn (Z.Y.); shengjiangkun@nint.ac.cn (J.S.); mawuying@nint.ac.cn (W.M.); dongguantao@nint.ac.cn) (G.D.); jinjunshan@nint.ac.cn (J.J.)

**Keywords:** CMOS image sensors (CISs), proton, dark signal distribution, theoretical, experimental

## Abstract

Four-transistor (T) pinned photodiode (PPD) CMOS image sensors (CISs) with four-megapixel resolution using 11µm pitch high dynamic range pixel were radiated with 3 MeV and 10MeV protons. The dark signal was measured pre- and post-radiation, with the dark signal post irradiation showing a remarkable increase. A theoretical method of dark signal distribution pre- and post-radiation is used to analyze the degradation mechanisms of the dark signal distribution. The theoretical results are in good agreement with experimental results. This research would provide a good understanding of the proton radiation effects on the CIS and make it possible to predict the dark signal distribution of the CIS under the complex proton radiation environments.

## 1. Introduction

Thanks to their low power consumption, high levels of integration, low noise, low cost, etc., pinned photodiode (PPD) CMOS image sensors (CISs) are widely used many scientific fields, such as star tracking, space remote sensing and medical imaging [1,2,3]. However, when applied in these applications, they would be seriously damaged by particles or rays, leading to image quality degradation or even functional failure. The dark signal distribution is one of the most important parameters of the CIS. The proton radiation would have great effects on the dark signal distribution of the CIS in space radiation environments. In depth analysis of the proton radiation effects on dark signal distribution of PPD CMOS image sensors is very important. 

Many studies have been dedicated to the proton radiation effects on the dark signal distribution of the CIS. Beaumel et al. [4] have done some experiments to investigate the Cobalt-60, proton and electron radiation effects on the dark signal distribution of the CIS. However, the total ionizing dose (TID) and displacement damage dose (DDD) effects were presented separately. Inguimbert [5] and Raine et al. [6] have used Greant4 to simulate the proton radiation effects on the dark signal distribution of the CIS, but the TID effects were not considered. Gilard [7] and Zheng et al. [8] have reported a method to predict the dark signal distribution of the CIS after proton radiation based on the empirical fitting of experiments data. However, the device structures were not considered which make it difficult to analyze the physical mechanisms of the degradation. Virmontois et al. [9,10,11] have done many works to investigate the proton and neutron radiation effects on the dark signal distribution of the CIS with different structures. The model, based on experimental data, was established to analyze the physical mechanisms. However, the TID effects on the dark signal distribution were often neglected. Sometimes, the TID effects can be neglected, especially when proton energy is higher. However, when the proton energy is lower, the TID effects cannot be neglected. For example, the TID of 3 MeV proton is more than 200 krad(Si) [12] when the fluence is about 1 × 10^11^ p/cm^2^. In order to analyze the mechanisms of dark signal distribution induced by the proton radiation and provide basis of theories and experimental techniques of the CIS radiation damage evaluation, proton radiation effects on dark signal distribution of CIS are studied.

In this paper, the proton radiation effects on the dark signal distribution of 4T PPD CISs are investigated with experimental and theoretical methods. Both TID and DDD effects are considered. Two CISs (with the same code) were irradiated by 3 MeV and 10 MeV protons at the EN Tandem Van De Graaff accelerator, Peking University, Beijing, China. The dark signal distributions are measured pre- and post- radiation. The theoretical methods of dark signal distribution pre- and post-radiation are presented. The theoretical results are in good agreement with experimental results. The degradation mechanisms of the CISs induced by TID and DDD effects are analyzed. 

## 2. Experimental Details

The two image sensors tested in experiments are 4T-PPD CIS using 11 µm pitch high dynamic range pixels with the same code. They are manufactured in the standard 0.18-µm CIS technology and the image array consists of 2048 × 2048 pixels. The sensor operates in electronic rolling shutter and features an extremely low temporal noise of 1.47e^−^. The CIS have on-chip 12-bit column-parallel analog to digital converter. The thickness of the glass windows is about 1.1 mm. High sensitivity combined with very low noise and low dark current makes it perfect for scientific applications.

**Figure 1:**
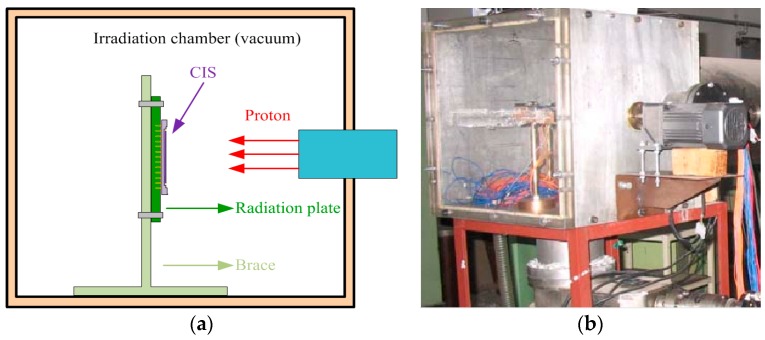
Experimental setup for the CIS proton radiation test: (**a**) schematic diagram of the experiment; and (**b**) photo of the irradiation chamber.

The proton radiation experiments were carried out at the EN Tandem Van De Graaff accelerator in the state key laboratory of nuclear physics and technology, Peking University, Beijing, China. Experimental setup for the CIS proton radiation test is shown in Figure 1. The proton radiation experiment conditions and samples are presented in Table 1. The samples are unbiased with all pins grounded during proton irradiation. Device #1 was exposed to 3 MeV protons and device #2 was exposed to 10 MeV protons. The CISs are measured at the accumulated fluence of 1 × 10^10^, 5 × 10^10^, and 1 × 10^11^ p/cm^2^ within 2 h after each irradiation step. The glass windows of samples are taken off before proton radiation to void most proton energy being absorbed by glass windows. The proton radiation experiments are performed at room temperature. Air-conditioning is used to keep the test chamber at room temperature during measuring and irradiating.

## 3. Experimental Results

During the proton radiation, the devices suffer both ionizing and non-ionizing damage, leading to the degradation of image quality. The TID effects lead to the dark signal of many pixels increasing. DDD effects lead to the dark signal of some pixels increasing and the appearance of dark signal spikes. Dark signal distribution is the histogram of dark signal of each pixel of the CIS. It is one of the most important parameters to evaluate the degradation degree of the CIS after proton radiation.

**Figure 2:**
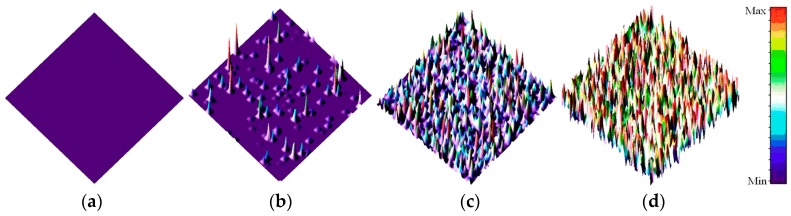
3-D surface plot of dark images from CIS (#1) After 3 MeV proton radiation (integration time: 61.56 ms): (**a**) before radiation; (**b**) proton fluence: 1 × 10^10^ p/cm^2^; (**c**) proton fluence: 5 × 10^10^ p/cm^2^; and (**d**) proton fluence: 1 × 10^11^ p/cm^2^.

**Figure 3:**
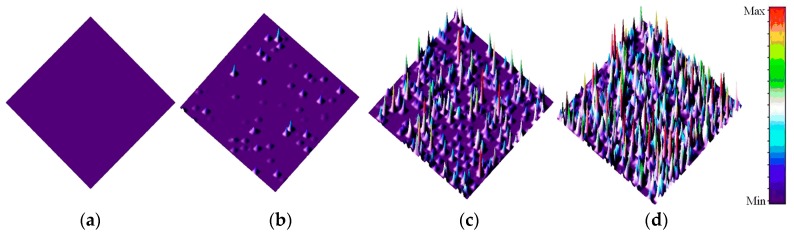
3-D surface plot of dark images from CIS (#1) After 10 MeV proton radiation (integration time: 61.56 ms): (**a**) before radiation; (**b**) proton fluence: 1 × 10^10^ p/cm^2^; (**c**) proton fluence: 5 × 10^10^ p/cm^2^; and (**d**) proton fluence: 1 × 10^11^ p/cm^2^.

Figure 2 shows the 3-D surface plot of the dark images from CIS (#1) after 3 MeV proton radiation (integration time: 61.56 ms). Figure 3 shows 3-D surface plot of dark images from CIS (#2) after 10 MeV proton radiation (integration time: 61.56 ms). In Figure 2 and Figure 3, one can see that the dark signal of CISs is extremely damaged after proton radiation. The damage degree of dark images increases with increasing of proton fluence. At the same fluence, the damage degree of the dark images radiated by 3 MeV proton is higher than those radiated by 10 MeV proton. Moreover, many “hot pixels”, which are also called dark signal spikes, appear after proton radiation. These spikes are induced by the DDD effects of the proton. DDD effects would induce bulk defects in space charge region (SCR) of the CIS. These defects can become the generation and recombination of electron–hole pairs, the carrier trapping, the compensation of donors or acceptors, and the tunneling of carriers which would induce dark signal spikes [13]. Dark signal spikes lead to the image quality degradation or even functional failure. 

**Figure 4:**
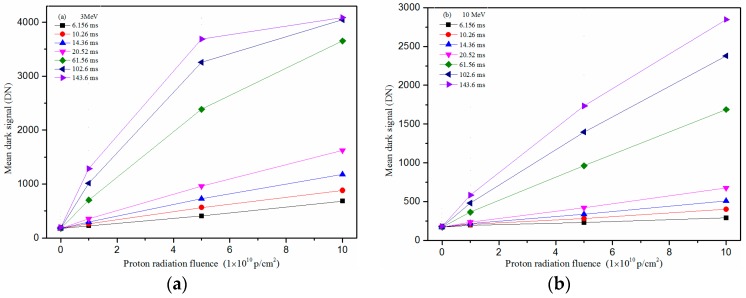
Mean dark signal versus proton fluence at different integration time: (**a**) proton energy: 3 MeV; and (**b**) proton energy: 10 MeV.

**Figure 5:**
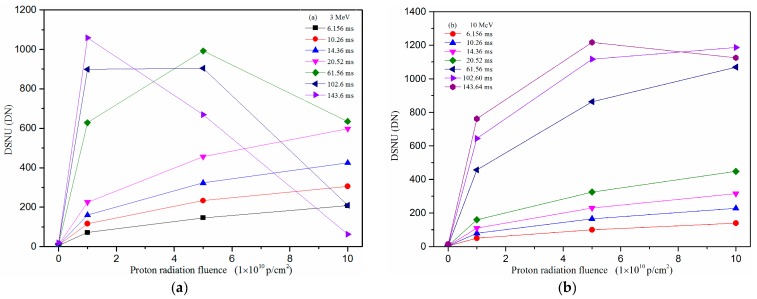
DSNU versus proton fluence at different integration time: (**a**) proton energy: 3 MeV; and (**b**) proton energy: 10 MeV.

Figure 4 shows the mean dark signal versus proton fluence at different integration times and Figure 5 shows the dark signal non-uniformity (DSNU) versus proton fluence at different integration times. In Figure 4, one can see that the mean dark signal increases with increasing of proton fluence. When the integration time is higher, the tendency of the dark signal increase is decreasing. In Figure 5, one can see that the DSNU decreases with increasing of the proton fluence when the integration time is higher. Both phenomena are because some of the pixels, which are damaged by protons, have reached saturation.

**Figure 6:**
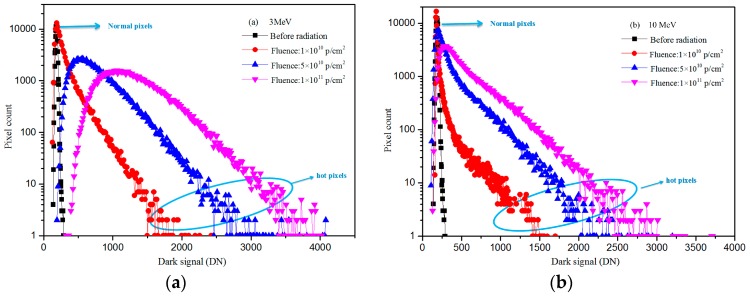
Dark signal distributions of CISs after proton irradiation: (**a**) proton energy: 3 MeV; and (**b**) proton energy: 10 MeV.

Figure 6 shows dark signal distribution of CISs after proton radiation: (a) proton energy of 3 MeV; and (b) proton energy of 10 MeV. In Figure 6, one can see that some of the pixels are not affected by proton when the fluence is lower. With increasing of proton fluence, most of the pixels are influenced and dark signal increases remarkably.

## 4. Theoretical Results

Dark signal stands for the output signal when the CIS is not exposed to light. It is one of the most important parameters for the CIS. For an ideal CIS, no dark signal can be detected because the free charge carrier generation is zero. However, dark current generation can also be dependent on the silicon used, thus devices from different batches can exhibit different dark current, and can lead to signal being detected even without light. 

**Figure 7:**
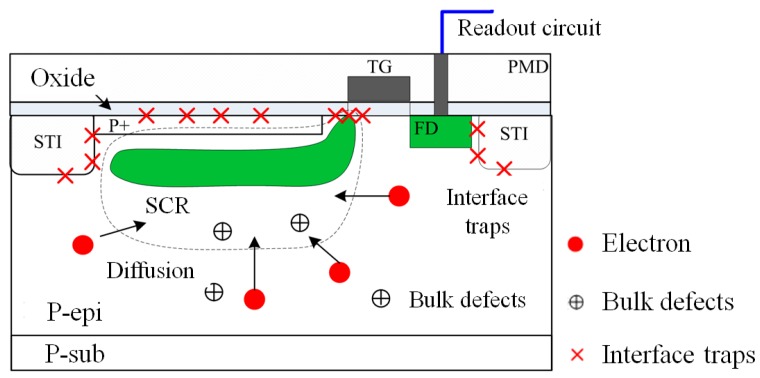
Main defects leading to dark current increase after proton radiation.

After proton radiation, many interface traps and bulk defects are generated in the CIS (show in the Figure 7), which lead to the dark current increase. The interface traps, which are generated in the shallow trench isolation (STI), pre-metal dielectric (PMD), and transfer gate (TG) produce surface leakage current. The bulk defects, which are generated in SCR, produce bulk current [13,14]. Moreover, some minority carriers can be diffused through SCR and then collected and readout. It is known as the bulk diffusion current. Compared with bulk current and surface leakage current, the bulk diffusion current can be neglected. Therefore, after proton radiation, the dark current increase mainly includes the bulk current (which is produced by DDD effects) and surface leakage current (which is produced by TID effects). According to the relationship between the dark current and dark signal, we can get that the degradation mechanism of dark signal is the same as dark current.

The nuclear elastic, nuclear inelastic and nuclear Coulombic scattering induce displacement damage. The TID is induced by ionizing interaction. The nuclear elastic and nuclear inelastic lead to a dark signal distribution that looks like a Gamma distribution. The TID and nuclear Coulombic scattering can lead to a dark signal distribution that looks like a Gaussian distribution [15]. It is dependent on the pixel size, depleted volume, cross section of interaction and the fluence of particles. Therefore, the dark signal distribution of the CIS after proton radiation is equal to the convolution of these two distributions.

**Figure 8:**
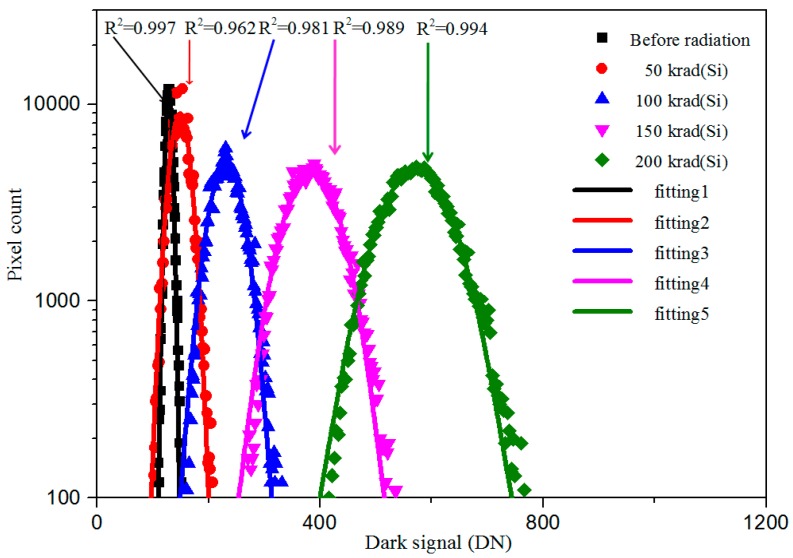
Dark signal distribution of the CIS after gamma radiation.

Figure 8 shows the dark signal distribution of the CIS after gamma radiation. The radiation dose rate is about 50 rad(Si)/s. The CIS was unbiased during irradiation. In Figure 8, one can see that the mean dark signal and DSNU increase with increasing of the TID. In addition, the dark signal distribution looks like a Gaussian distribution. After gamma rays radiation, mostly of the pixels are affected because of the cross section of gamma rays in Si is high. Therefore, the dark signal distribution looks like a Gaussian distribution. Gaussian function is used to fit the dark signal distribution, and the goodness-of-fits are, respectively, R^2^ = 0.997, R^2^ = 0.962, R^2^ = 0.981, R^2^ = 0.989 and R^2^ = 0.994 for the CIS after radiated by γ rays with TID 0, 50, 100, 150 and 200 krad(Si).

**Figure 9:**
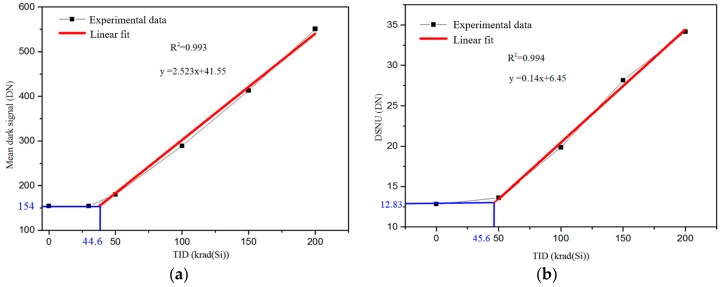
Mean dark signal and DSNU of CIS verse TID: (**a**) mean dark signal; and (**b**) DSNU.

Figure 9 shows the mean dark signal and DSNU of the CIS versus TID. In Figure 9, one can see that there is a threshold of the TID effects (TID_th_) on dark signal and DSNU of the CIS: When the TID greater than TID_th_, the mean dark signal and DSNU increase appears proportional to the TID. Line fitting of the TID and the dark signal and DSNU are performed and the goodness-of-fits are about R^2^ = 0.993 (for the mean dark signal) and R^2^ = 0.994 (for the DSNU). The TID_th_ of the dark signal is about 44.0 krad(Si) and the TID_th_ of DSNU is about 45.6 krad(Si). Therefore, at a given TID, we can calculate the dark signal and DSNU of this kind of CIS, which stands for the expectation and the standard deviation of dark signal distribution. For example, if the TID is 30 krad(Si), the TID radiation effects on the CIS can be neglected; if the TID is 120 krad(Si), the dark signal and DSNU of the CIS would become 339.7 DN and 23.25 DN. 

The Gamma distribution has two parameters: shape parameter and scale parameter. Combined with the physical progress, we can find than shape parameter is the same as the effective interactions per pixel and the scale parameter is the same as the mean dark signal induced by one interaction. At a given fluence and energy, interactions per pixel *µ*_0_ are given as:

(1) $$\mu_{0}=\sigma N_{\mathrm{at}}V_{\mathrm{dep}}\varphi$$

where *σ* is the mean interaction cross-section; *N*_at_ is the silicon atomic density; *V*_dep_ is the depleted volume in the pixel; and φ is the fluence per cm^2^. When the proton energy is different, while *µ*_0_ is the same, the mean dark signal induced by the one interaction is different. To make it the same, the effective interactions per pixel *µ*_1_ are carried out and given as:

(2) $$\mu_{1}=\mu_{0}\times \frac{DDD_{1}}{DDD_{0}}$$

where *µ*_0_ is the interactions per pixel when the energy is *E*_0_; *DDD*_1_ is the displacement damage dose when the energy is *E*_1_; and *DDD*_0_ is the displacement damage dose when the energy is *E*_0_.

**Figure 10:**
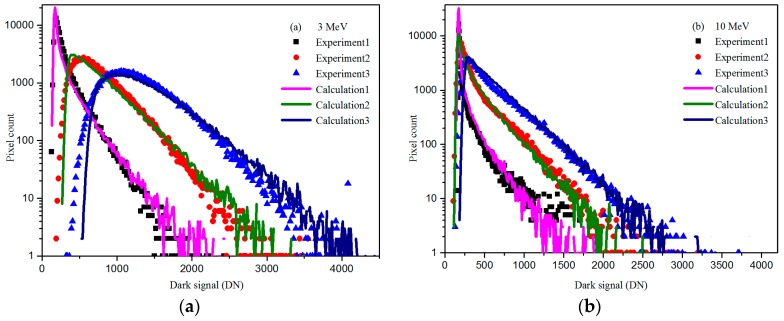
Experimental (point) and calculated (lines) distributions for CISs after proton radiation: (**a**) 3 MeV; and (**b**) 10 MeV.

A 3D Monte Carlo code, Geant4 [16], is used to calculate the TID and DDD of the proton in CIS. The geometry of the CIS has great effects on the calculated results. In this work, the simulation model is built according to the real pixel geometry, material and doping concentration. The radiation dose of 3 MeV and 10 MeV proton at different fluence in the STI of the CIS are calculated. The effective interactions per pixel are calculated according to Equation (2). The calculated results are shown in the Table 2. In Table 2, one can see that the effective interactions per pixel is about 1 when the CIS is radiated by10 MeV proton with fluence of 1 × 10^11^ p/cm^2^. The dark signal induced by protons can be separated into two signals [17]:

(3) $$\Delta \mu_{\mathrm{dark}}=\Delta \mu_{\mathrm{dark},\mathrm{TID}}+\Delta \mu_{\mathrm{dark},\mathrm{DDD}}$$

where ∆*µ*_dark_ is the dark signal increase; ∆*µ*_dark,TID_ is the dark signal increase induced by TID effects; and ∆*µ*_dark,TID_ is the dark signal increase induced by DDD effects. The synergistic effects of TID and DDD are not considered at this time. Combined with the TID experimental results, we can get the ∆*µ*_dark,DDD_ of the CIS after radiated by 10 MeV proton with fluence of 1 × 10^11^ p/cm^2^ which is scale parameter of the Gamma distribution. Therefore, we can get the dark signal distribution of the CIS induced by TID and DDD. Combining these two distributions, we can get the distribution of CIS after proton radiation. Figure 10 shows the experimental (point) and calculated (lines) distributions for CISs after proton radiation. In Figure 10, one can see that the experimental results are in good agreement with theoretical results. Therefore, we can use this way to predict the dark signal distribution of the CIS under the complex radiation environments.

## 5. Conclusions

Dark signal distribution in the PPD CIS affected by proton with different fluence and energy are investigated. The CIS is manufactured in the standard 0.18-µm CIS technology and the image array consists of 2048 × 2048 pixels. The experiments were carried out using 3 MeV and 10 MeV protons at the EN Tandem Van De Graff accelerator. The proton flux is about 3.7 × 10^7^ (p/cm^2^·s) and the accumulated fluence are 1 × 10^10^, 5 × 10^10^, and 1 × 10^11^ p/cm^2^. To analyze the TID effects of the proton on CISs, Colbalt-60 gamma-ray radiation experiments were also carried out. The dose rate is about 50 rad(Si)/s with the same condition as proton radiation experiments. The dark signal distribution of the CIS is measured pre- and post-radiation.

The theoretical methods of proton radiation induced dark signal distribution degradation are established according to the real interaction of the proton. Proton radiation effects include TID and DDD effects, and both lead to the dark signal distribution degradation, which makes it difficult to analyze the degradation mechanisms. TID effects would influence nearly all of the pixels of CISs and lead to a dark signal distribution increase that looks like a Gaussian distribution. The expectation and standard deviation of this distribution can be calculated according to the Colbalt-60 gamma-ray radiation experiments. DDD effects lead to a dark signal distribution increase that looks like a Gamma distribution, and the shape parameter and scale parameter can be calculated according to the proton radiation experiments and Geant4 simulation. After proton radiation, the dark signal distribution increase could be calculated by convolution of these two distributions. The experimental results are in good agreement with calculated results. 

## Figures and Tables

**Table 1:** Proton radiation experiment conditions and samples.

CIS Number	Bias Condition	Proton Energy (MeV)	Proton Flux (p/cm^2^/s)	Proton Fluence (10^10^p/cm^2^)
1#	Unbiased	3	3.75 × 10^7^	1,5,10
2#	Unbiased	10		1,5,10

**Table 2:** Calculated parameters.

Proton Energy (MeV)	Proton Fluence (10^10^p/cm^2^)	TID (krad(Si))	Effective Interactions per Pixel
3	1	23.5	0.24
3	5	117.5	1.2
3	10	235.0	2.4
10	1	9.6	0.1
10	5	47.9	0.5
10	10	95.8	1.0
